# Negligible hormonal response following dehorning in free-ranging white rhinoceros (*Ceratotherium simum*)

**DOI:** 10.1093/conphys/coaa117

**Published:** 2020-12-29

**Authors:** Samuel G Penny, Rachel L White, Lynne MacTavish, Dawn M Scott, Angelo P Pernetta

**Affiliations:** 1 Ecology, Conservation and Zoonosis Research Group, School of Pharmacy and Biomolecular Sciences, University of Brighton, Brighton BN2 4GJ, UK; 2 Stand 3300, Thabazimbi Road, Rustenberg, Northwest Province, South Africa; 3 School of Life Sciences, Keele University, Newcastle-under-Lyme ST5 5BG, UK

**Keywords:** Anti-poaching, glucocorticoids, progesterone, stress, testosterone, wildlife management

## Abstract

The white rhinoceros (*Ceratotherium simum*) is experiencing unsustainable poaching losses fuelled by a demand for horn. Increasingly, private and state reserves are dehorning their rhinoceros populations in an attempt to reduce poaching pressure. Rhinoceroses use their horns in social interactions as well as during resource access and so its partial removal as part of reserve management practices may adversely influence these behaviours. Physiological stress can correlate with animal welfare, reproductive state and health and thus acts as a useful indicator of these parameters. To establish whether dehorning causes a physiological stress response, glucocorticoid and gonadal steroid profiles of free-ranging white rhinoceroses were determined through the collection and analysis of faecal steroid metabolites before and after dehorning. Faecal corticoid profiles were not influenced by the number of occasions a rhinoceros had been dehorned or by the number of days that had elapsed since dehorning. Furthermore, there was no apparent suppression in the concentrations of testosterone or progesterone metabolites in males and females, respectively, after exposure to multiple dehorning procedures. These findings should increase wildlife managers’ confidence that dehorning does not negatively impact white rhinoceros physiology as measured hormonally.

## Teaser

Private reserves and national parks in southern Africa are increasingly dehorning their rhinos. This study monitored changes in the faecal hormone metabolites of white rhinos subject to this procedure. The findings indicate that dehorning had a negligible impact on hormone metabolite profiles and that dehorning did not cause a long-term physiological stress response in rhinos.

## Introduction

Rhinoceros species (from here on ‘rhino’) require immediate actions to save them from unsustainable poaching losses (Taylor *et al.*, 2017; Ferreira *et al.*, 2018). A rise in demand for rhino horn in East Asia has resulted in the deaths of over 8000 African rhinos during the 2010s (Vigne and Martin, 2018; Knight, 2020). Field responses include increases in security and law enforcement (Haas and Ferreira, 2018), the strategic translocation or herding of rhinos from areas of high risk to areas of low risk (Penny *et al.*, 2019) and dehorning (Knight, 2020). Faced with high security costs, private reserves and national parks in southern Africa are increasingly dehorning their rhinos as an attempt to reduce poaching (Rubino and Pienaar, 2018; Knight, 2020). However, in many cases, these interventions are performed as a crisis response and lack scientific evaluation of its effectiveness at reducing poaching or as investigated here, its biological impact (Lindsey and Taylor, 2011; du Toit and Anderson, 2013). Research on whether dehorning causes an endocrine stress response in free-ranging white rhinos can aid the implementation and design of conservation strategies by identifying whether there is a negative physiological impact.

Dehorning, also known as horn trimming, refers to the controlled removal of a portion of a rhino’s horn by veterinary procedure (Badenhorst *et al.*, 2016). Dehorning in South Africa only became common as an anti-poaching tool from 2008, after poaching rates had dramatically increased (Lindsey and Taylor, 2011). Until recently, dehorning in the country was predominately performed on smaller populations in private reserves, rather than provincial and federal run reserves (Taylor *et al.*, 2014). However, the current poaching epidemic has seen dehorning become an increasingly common conservation intervention in areas that suffer high poaching rates as the cost of effective security rises (Lindsey and Taylor, 2011; Taylor *et al.*, 2014). In addition to its use as an anti-poaching tactic, dehorning is also practised to prevent a rhino from injuring itself or another individual (Patton *et al.*, 2018) and to harvest or stockpile horn for financial gain (Taylor *et al.*, 2014). There are approximately 400 private rhino owners in South Africa and a survey of 52 respondents indicated that 37% dehorned their rhinos (Taylor *et al.*, 2014). Furthermore, several larger national parks have committed to dehorn at least some of their rhinos (Knight, 2020), with Kruger National Park beginning the selective dehorning of females in 2019 (SANParks, 2019) and Pilanesberg National Park dehorning their entire rhino population in 2020 (Sishi and Cocks, 2020).

Rhino horns are made entirely of keratin and are not attached to the skull; this makes them different to the keratin-and-bone horns found in the Bovidae or the solid bone horns of the Cervidae (Hieronymus *et al.*, 2006). Rhino horn is thus neither enervated nor vascularized and grows continuously throughout a rhino’s life, increasing in both diameter and length (Pienaar and Hall-Martin, 1991). In dehorned white rhinos, this equates to a height increase of ~7 cm per year for the anterior horn and 3 cm per year for the posterior horn (Kock and Atkinson, 1993; Rachlow and Berger, 1997). This leads to a mean increase of 0.861 kg per year in male white rhino horns and 0.635 kg per year in females (Ververs, 2018). Dehorning, where applied, therefore requires rhinos to undergo repeat procedures throughout their lifetime to be effective. The frequency of repeat dehorning for anti-poaching purposes depends on the management criteria, the poaching threat and the costs involved, but usually a time of 12–36 months is advocated between instances (Rachlow and Berger, 1997; Lindsey and Taylor, 2011).

Exposure towards anthropogenic stressors can lead to a physiological stress response in vertebrates (Dickens *et al.*, 2010; Dickens and Romero, 2013). Early physiological effects include heightened cardiovascular tone (e.g. via an increase in heart or breathing rate), increased energy mobilization (e.g. the transfer of energy from stores to exercise muscle) and a decline in proceptive and receptive sexual behaviours (e.g. erection loss) (Sapolsky *et al.*, 2000). In the short term, an acute stress response acts to increase an animal’s chances of surviving hostile or challenging conditions (Dickens *et al.*, 2010). However, in the long term, if the perturbation is chronic or a consecutive series of acute stressors, then the stress response can become maladaptive and deleterious to an animal’s health or reproductive fitness, a condition sometimes termed distress (Dickens *et al.*, 2010; Linklater *et al.*, 2010; McCormick and Romero, 2017). In the long term, pathologies may arise through processes such as cardiovascular change (e.g. hypertension or myocardial infarction; Rupp, 1999), immunosuppression (e.g. reduced cell-mediated immune responses; Dhabhar and McEwen, 1997) and reproductive suppression (e.g. reduced conception and increased abortion rates; Young *et al.*, 2006).

Glucocorticoids function as mediators of the physiological stress response, making them an ideal marker for how rhinos respond to a known or potential stressor (Palme, 2019), including in response to wildlife management techniques such as immobilizations and translocations (Kock *et al.*, 1990; Linklater *et al.*, 2010; Capiro *et al.*, 2014; Göttert *et al.*, 2015; Yang *et al.*, 2019). If the stress response is acute, glucocorticoids inhibit their own production and return to normal levels (Sapolsky *et al.*, 2000). In comparison, chronic stressors can disrupt the negative feedback of glucocorticoids, resulting in their prolonged elevation (Romero, 2004). During longer-term monitoring of the stress response, it can also be useful to observe other indicators of health such as androgens and progestogens; gonadal steroids with functions in male and female rhino reproduction, respectively (Kretzschmar *et al.*, 2004; Linklater *et al.*, 2010; Roth *et al.*, 2018).

For example, in a study of translocated white rhinos, the subjects underwent a sustained rise in glucocorticoid concentrations between two and five times of pre-capture concentrations during their first 17 days of confinement, as well as decreases in gonadal steroids (Linklater *et al.*, 2010). Throughout the remaining 8 weeks of captivity, the glucocorticoid concentrations of males returned to pre-capture concentrations but androgen concentrations decreased further. In comparison, the glucocorticoid concentrations of females remained high throughout captivity, with progestogen concentrations remaining suppressed. Thus, looked at over longer periods, declining concentrations of glucocorticoids does not necessarily indicate an absence of a chronic or longer-term physiological stress response, and additional biomarkers should be considered (Linklater *et al.*, 2010).

Faeces have been validated as a suitable medium to monitor changes in white rhino glucocorticoid and gonadal steroid metabolites by several studies (e.g. Hindle and Hodges, 1990; Brown *et al.*, 2001; Kretzschmar *et al.*, 2004; Riato, 2007). Furthermore, faecal samples can be collected non-invasively without disturbing the animal (Kretzschmar *et al.*, 2004) and can remain stable for several hours after defecation (Turner *et al.*, 2002). The peak clearance of rhino faecal corticoid metabolites occurs 24–48 hours after the production of native hormones, returning to baseline concentrations 4 days after (Riato, 2007). Testosterone and progesterone also do not show peak clearances until after 24 hours of production (Hindle and Hodges, 1990; Kretzschmar *et al.*, 2004). This makes faeces more appropriate for studies of large free-ranging mammals than blood sampling if pre-capture responses are desired (Palme, 2019). Furthermore, as faecal metabolites represent the cumulative secretion of steroidogenic activity over several hours, it acts to diminish the circadian and pulsatile flux found in measures of circulating hormones (Whitten *et al.*, 1998).

Research on the physiological consequences of dehorning is limited to a single study of intensively reared female white rhinos, which experienced a spike in faecal glucocorticoids in the first 4 days after the procedure (Badenhorst *et al.*, 2016). However, it is unknown whether the physiological effects extend beyond this period, affect both sexes, or occur in free-ranging populations, leading to calls for further study (Ververs, 2018). This study determined whether dehorning causes a physiological stress response in free-ranging white rhinos. The primary research questions were (i) whether dehorning caused a detectable change in glucocorticoid or gonadal steroid metabolites and (ii) whether the number of times a rhino was dehorned influenced the rate of this change.

## Materials and methods

### Study population

Faecal samples were collected from 16 white rhinos from a 4932-ha privately owned game reserve in Northwest Province, South Africa (see Penny *et al.*, 2020 for further site details). The rhinos received limited husbandry and veterinary care and had a natural breeding strategy. No supplementary feeding occurred but the rhinos had access to several artificial mineral licks and water sources. Thus, despite being fenced, the population met the African Rhino Specialist Group criteria for a wild population (Leader-Williams *et al.*, 1997). Dehorning began at the site in 2014 (Table 1). Rhinos were classed as subadults from maternal independence until they reached socio-sexual maturity (Table 1). This is when males become solitary and/or territorial at 10–12 years old and at ~7 years old in females after the birth of their first calf (Shrader and Owen-Smith, 2002).

**Table 1:** Parameters of the rhinos monitored throughout the study

ID	Sex	Age	Dehorning dates			Group
			1	2	3	
1	F	SA/A	20 October 2014^a^	02 June 2016		I
2	F	A	20 October 2014^a^	02 June 2016		I
3	M	A	20 October 2014^a^	02 June 2016	21 July 2017	I
4	M	SA/A	20 October 2014^a^	02 June 2016	06 June 2017	I
5	M	SA	27 October 2014^a^	02 June 2016	06 June 2017	I
6	M	C/SA	20 October 2014^a^	02 June 2016	06 June 2017	I
7	M	SA	27 October 2014^a^	02 June 2016	06 June 2017	I
8	M	SA	20 October 2014^a^	02 June 2016	06 June 2017	I
9	F	A	20 October 2014^a^	20 April 2017		II
10	F	A	20 October 2014^a^	20 April 2017		II
11	F	A	20 October 2014^a^	20 April 2017		II
12	M	A	13 September 2015^a^	06 June 2017		II
13	M	C	20 April 2017			II
14	M	C	20 April 2017			II
15	M	C	20 April 2017			II
16	M	C	20 April 2017^a^			II

Rhinos in Group I were dehorned on 02 June 2016; rhinos in Group II were not dehorned on this date or throughout 2016.

Sex classes: F indicates female and M indicates male. Age classes: A indicates adult, SA indicates subadult and C indicates calf.

^a^ No samples were collected prior to this date.

Project methods were reviewed and approved by the game reserve managers and abide by the ethical guidelines set by the American Society of Mammalogists (Sikes and Animal Care and Use Committee of the American Society of Mammalogists, 2016). Ethical approval was also granted by the Animal Welfare and Ethics Review Board of the University of Brighton (Ref: 2018-1127). Permission was granted by rhino owner D. MacTavish to conduct research at the site and monitor the rhinos in response to dehorning. Prior to each dehorning, the necessary permits were obtained from North West Department of Economic Development, Environment, Conservation and Tourism (DEDECT) by the reserve manager (information available on request). The representatives of DEDECT were present at each dehorning to confirm that all official guidelines were followed throughout the procedure. Dehorning personnel consisted of at least two qualified veterinarians, several assistants and the reserve management staff. Immobilization occurred by shooting a dart into the rump or the gluteal muscles of a rhino from a helicopter. The immobilization cocktail consisted of intramuscular etorphine hydrochloride (M99), azaperone (Stresnil) and hyaluronidase with the dose varied according to animal body size (see Morkel and Nel, 2019). Following immobilization, rhinos were placed in sternal or lateral recumbency and equipped with ear and eye covers to reduce sensory input. Once the rhinos were recumbent, respiratory depressant effects were partially reversed with butorphanol and their breathing rates were monitored (Morkel and Nel, 2019). The anterior and posterior horns were then trimmed with chainsaws and the edges smoothed with a disk sander. Care was taken to cut above the skin-horn interface (9–11 cm) to prevent damage to the growth plate and sinuses. Finally, the effects of the opioids were reversed with intravenous naltrexone. The complete dehorning procedure, from initial darting to full recovery took ~15–20 minutes per rhino.

### Study design

Corticosterone, testosterone and progesterone metabolite concentrations were measured from faecal samples; the data per rhino were then analysed over time to evaluate whether metabolite levels changed. As these hormones are heavily metabolized before excretion, the assays rely on the cross-reactivity of their metabolites (Palme, 2019). This makes it more accurate to refer to the measured concentrations as faecal corticosterone metabolites (FCMs), faecal androgen metabolites (FAMs) and faecal progestogen metabolites (FPMs) rather than as native hormones.

To investigate whether rhinos exhibited a short-term physiological stress response to dehorning, FCM concentrations were measured in eight rhinos up to 1 week before a dehorning procedure on 02 June 16 (range, 0–117 hours before; mean, 15 hours; *n* = 8) and compared with the FCMs of samples collected in the week after dehorning (range, 24–166 hours after; mean, 80 hours; *n* = 8). No samples were collected in the first 24 hours after the procedure to account for the time lag between stressor and response (Riato, 2007) and changes in FAMs and FPMs were not monitored. This was the second time that the rhinos had undergone a dehorning (Group 1, Table 1), as the rhinos had undergone a prior procedure between 584 and 591 days earlier.

To investigate whether rhinos experienced a longer-term physiological stress response to dehorning, the FCM profiles of eight rhinos (Group 1) monitored between 1 and 142 days after the procedure (on 02 June 2016) were contrasted against the FCM profiles of the eight rhinos that had not recently been dehorned (Group II) over the same time period. In total, 89 samples were analysed (2–8 per rhino). Changes in FAMs and FPMs were not monitored. Data for both groups were collected over the same contiguous period from 03 June 2016 until 22 October 2016, with all rhinos exposed to similar environmental conditions and comparable levels of forage availability and disturbance (e.g. from anti-poaching units, vehicles and people).

Finally, to investigate whether rhinos exhibited a physiological stress response to multiple dehorning events, FCM, FPM and FAM profiles were related to the number of dehorning procedures that each rhino had been subjected to and the number of days that had passed since their first dehorning. FCMs were analysed from 15 rhinos (IDs 1–15, Table 1) collected over a 516-day monitoring period from 24 May 2016 to 22 October 2017. The monitored period did not begin until 110 days or greater after any rhino’s first dehorning. Rhinos had been dehorned between one and three times each by the end of the monitored period. In total, 143 samples were analysed (1–10 per dehorning event per rhino). FPMs were analysed from five female rhinos (IDs 1, 2, 9–11, Table 1) collected over a 482-day monitoring period from 24 May 2016 to 17 September 2017. The monitored period did not begin until 580 days or greater after any female rhino’s first dehorning. The female rhinos had been dehorned twice by the end of the monitored period. FPMs were not analysed from samples collected during pregnancy, which was calculated by back-counting the average white rhino gestation length of 495 days from parturition (Linklater, 2007). In total, 35 samples were analysed (1–9 per dehorning event per rhino). FAMs were analysed from seven male adult and subadult rhinos (IDs 3–8, 12, Table 1) collected over a 516-day monitoring period from 28 May 2016 to 22 October 2017. The monitored period did not begin until 258 days or greater after any rhino’s first dehorning. Rhinos had been dehorned between two and three times each by the end of the monitored period. In total, 89 samples were analysed (1–11 per dehorning event per rhino).

### Endocrine analysis

Samples were only collected if they could be matched to a specific individual. Faecal samples were collected from the centre of a dung pile or rectally from immobilized rhinos. Samples were sealed in airtight screwcap bijou bottles within 1 hour of defecation, kept cool and frozen within 4 hours of collection. An aliquot (0.150 ± 0.005 g) of each sample was weighed on a balance (VWR LPC-213) and then added to a solution of one-part ethanol (750 μl, 90%) to one-part distilled water (750 μl). Next, the mixture was agitated in a vortex shaker for 5 minutes (Fisherbrand Mini Vortex Mixer; 2.8 K rpm) to create a faecal slurry. The slurry was centrifuged for 15 minutes (SciSpin Mini Microfuge; 7000 rpm) and then 500 μl of the resulting supernatant was pipetted into a screwcap tube (Eppendorf; 2 ml) and stored at 4°C. To establish the dry weight of each sample, a second aliquot of faecal matter was air-dried and weighed until it stabilized.

Endocrine analyses were conducted in the UK at the University of Brighton with permission to import rhino faecal extract from South Africa granted from DEFRA (authorization no: ITIMP16/1052). Commercial enzyme immunoassay kits for corticosterone, progesterone and testosterone were purchased from Enzo Life Sciences (lot numbers: ADI-900-097, ADI-901-097, ADI-900-011, ADI-900-065). Samples were vortexed before analysis and then diluted into sample assay buffer to eliminate matrix interference. To determine optimal detection rates, serial dilutions of samples were made for each hormone. Dilutions were selected that fell within the most linear part of the standard curve and did not generate absorbance values beyond the highest standards (corticosterone: 20 000 pg/ml; testosterone: 2000 pg/ml; progesterone: 500 pg/ml). Samples were run in duplicate in 96-well microtiter plates in parallel with a series of known concentrations of target analyte. Once determined, initial concentrations were adjusted by the water content of their initial weight, to equal mass hormone per mass faecal dry weight (see Ezenwa *et al.*, 2012).

To calculate the percentage recovery of each hormone, undiluted faecal extracts were spiked with known concentrations of each hormone; these were then diluted in buffer and assayed alongside unspiked samples. A significant recovery of exogenous hormone was demonstrated for corticosterone (91%), testosterone (75%) and progesterone (78%). The dilutions that resulted in the greatest recovery of hormone were selected for further use. Additionally, the manufacturer had previously tested the corticosterone assay against white rhino faeces and reported a sample recovery of 93.9% (Enzo Life Sciences, 2015). Serial dilutions of faecal extracts were checked for and yielded parallelism between their binding inhibition curves and the hormone standards. Assay sensitivity for corticosterone was 7.90 pg/ml (*n* = 7 replicates), 2.41 pg/ml for testosterone (*n* = 4 replicates) and 10.79 pg/ml for progesterone (*n* = 2 replicates). Average intra- and inter-assay coefficients of variability were <16% for FCMs and <10% for FAMs and FPMs. The degree of cross-reactivity between each target metabolite and potential cross-reactants were conducted by the assay manufacturer (Enzo Life Sciences, 2015, 2016a,b).

### Statistical analysis

All analyses were two-tailed, and all alpha levels were set at 0.05. All statistical analyses were performed in R (version 3.5.1; R Core Team, 2018). For the analysis of short-term physiological stress, a Wilcoxon signed-rank test was run using the base R function to account for the small sample size and paired dataset (*n* = 8, obs. = 16). Generalized linear mixed models (GLMMs) with Laplace approximation were conducted for all other analyses using the ‘lme4’ package (Bates *et al.*, 2015). The GLMMs were fit with gamma distributions with log link functions to account for the distribution of the residuals and continuous predictor variables were centred and scaled. To account for the repeated measures design and the pooling of different ages and sexes, rhino identity was included as a random effect with each subject given the freedom to vary by the inclusion of a random intercept and random slope. Thus, directional physiological changes could still be detected even if subjects’ faecal metabolite concentrations started from different baselines or varied in their rate of change. Type-3 Wald χ^2^ tests were used to extract the significance of fixed effects using the ‘car’ package following checks of over-dispersion (see Thomas *et al.*, 2015). The conditional R^2^ value, which represents the variance explained by the entire model, was calculated using the delta method in the ‘MuMIn’ package (Johnson, 2014).

To investigate changes in FCM concentration over a longer 142-day period (*n* = 16, obs. = 89), two fixed effects were included in a GLMM: ‘Horn Change’ (whether or not a rhino was dehorned on Day 0) and ‘Sample Collection Date’ (days passed since Day 0). An interaction term was included to indicate whether ‘Horn Change’ had a differential impact over time. To investigate the changes in FCM (*n* = 15, obs. = 143), FPM (*n* = 5, obs. = 35) and FAM (*n* = 7, obs. = 89) concentrations following multiple dehorning procedures, two fixed effects were included in three GLMMS—‘Days Since First Dehorning’ and ‘Number of Dehorning Procedures’—that each rhino had been subject to at the time of sample collection. An interaction term between the two fixed effects was included to indicate whether the ‘Number of Dehorning Procedures’ had a differential impact over time. Rainfall varied over the 516-day monitored period, so season (with two levels: high rainfall and low rainfall) was included as a random effect and fit with a random intercept. However, it was excluded from the final models as its variance was estimated to be zero (FCMs and FAMs) or its inclusion resulted in a singular fit (FPMs) indicating the model had been over-fit. Additionally, the random slope for rhino identity was excluded from the final model of FAMs due to a further ‘singular fit’ warning.

## Results

### Short-term physiological response

**Figure 1:**
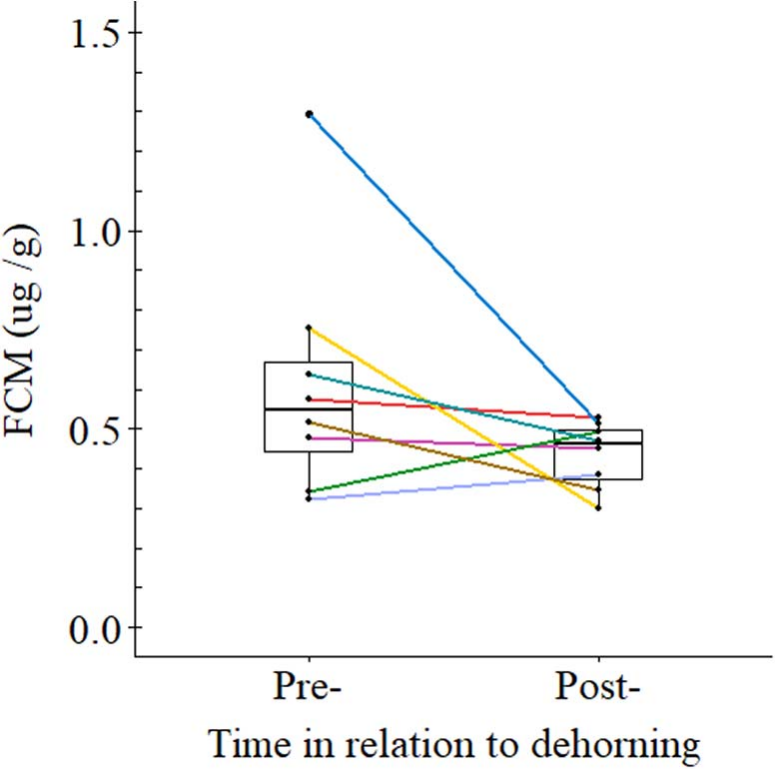
FCM concentrations sampled up to 1 week before and 1 week after a dehorning procedure (*n* = 8). Colours represent individual rhinos. Boxes indicate median and interquartile ranges

FCM concentrations of samples collected in the week prior to a dehorning procedure did not differ significantly to samples collected over the following week (Wilcoxon signed-rank: *V* = 29, *P*-value = 0.148, *n* = 8). The FCM concentrations of rhinos were a median 17.2% (range: −60 to 43%) lower after the procedure (median: 0.546 μg/g, *n* = 8) than those collected before dehorning (median: 0.460 μg/g, *n* = 8; Fig. 1).

### Longer-term physiological response

**Figure 2:**
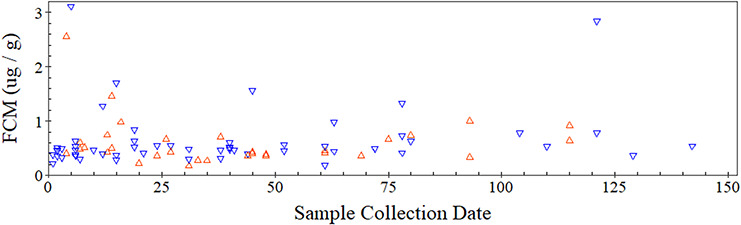
Variation in concentration of FCMs in rhinos that underwent a dehorning (Group 1 rhinos—orange) and those that did not (Group 2 rhinos—blue) over a 141-day monitored period. 0 indicates the day of dehorning; obs. = 89; *n* = 16.

FCM profiles monitored over a 141-day period did not differ significantly between the recently dehorned group of rhinos and the control group, as shown by the non-significant interaction term (*P* = 0.547; Table 1) illustrated in Fig. 2. Furthermore, when considering the terms of the individual fixed effects, FCM concentration was not influenced by sample collection date (*P* = 0.973; Table 2) nor was it influenced by which group a rhino was in (recently dehorned: mean, 0.642 ± SD 0.537 μg/g; control: 0.598 ± 0.446 μg/g; *P* = 0.934; Table 2).

**Table 2:** GLMM testing for the effect of time and dehorning treatment on FCMs over a 141-day period

Fixed effects	Estimate	Standard error	χ^2^	df	*P*-value
Sample collection date	0.015	0.212	0.005	1	0.943
Horn change	−0.06	0.156	0.173	1	0.677
Interaction (overall)	0.185	0.20	0.852	1	0.356

Horn change indicated whether or not rhinos were dehorned on Day 0. Sample Collection Date indicated the days passed since Day 0. Type-3 Wald χ^2^ tests were used to extract the significance of fixed effects. df indicated degrees of freedom.

*n* = 16, obs. = 89, conditional R^2^ = 34.3%.

### Response to multiple dehorning procedures

When considering the terms of the individual fixed effects, FCM concentrations were not significantly influenced by either the number of days since first dehorning (*P* = 0.110; Table 3) or by the number of dehorning procedures (*P* = 0.097; Table 3; first dehorning: mean, 0.613 ± 0.340 μg/g; second dehorning: 0.668 ± 0.506 μg/g; third dehorning: 0.460 ± 0.196 μg/g).

**Table 3:** GLMMs testing for the effect of the number of days since first dehorning and the number of dehorning procedures a rhino has been subject to on FCMs, FPMs and FAMs

Fixed effects	Estimate	St. error	χ^2^	df	*P*-value
FCMs					
Days since first dehorning	0.615	0.104	2.546	1	0.110
No. of dehornings (1 vs 2)	0.267	0.136			
No. of dehornings (2 vs 3)	−0.698	0.786			
No. of dehornings (overall)			4.666	2	0.097
Interaction (1 vs 2)	−0.274	0.160			
Interaction (2 vs 3)	0.095	0.558			
Interaction (overall)			2.949	2	0.229
FPMs					
Days since first dehorning	0.102	0.090	1.291	1	0.256
No. of dehornings	0.005	0.199	0.001	1	0.979
Interaction (overall)	0.110	0.135	0.659	1	0.417
FAMs					
Days since first dehorning	−1.329	0.072	15.495	1	<0.001^*^
No. of dehornings (1 vs 2)	−0.250	0.162			
No. of dehornings (2 vs 3)	−0.602	0.462			
No. of dehornings (overall)	-	-	4.003	2	0.135
Interaction (1 vs 2)	−0.549	0.171			
Interaction (2 vs 3)	0.372	0.400			
Interaction (overall)			12.517	2	0.002^*^

Type-3 Wald χ^2^ tests were used to extract the significance of fixed effects.

df indicates degrees of freedom and * indicates statistically significant.

FCMs: *n* = 15, obs. = 143, conditional R^2^ = 35.8%; FPMs: *n* = 5, obs. = 35, conditional R^2^ = 40.9%; FAMs: *n* = 7, obs. = 89, conditional R^2^ = 46.0%.

**Figure 3:**
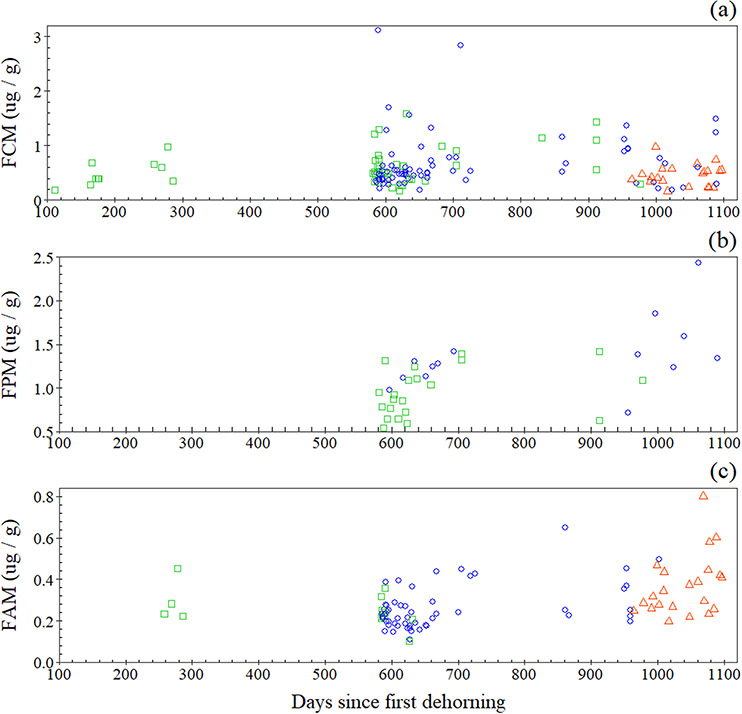
Variation in concentration of (a) FCMs, (b) FPMs and (c) FAMs following one (green squares), two (blue circles) and three (red triangles) dehorning procedures. FCMs: obs. = 143; *n* = 15; FPMs: obs. = 35, *n* = 5; FAMs: obs. = 89, *n* = 7.

The number of times a female rhino was dehorned did not influence the rate of change in FPM concentration, as shown by the non-significant interaction term (*P* = 0.417; Table 3; Fig. 3). Considered in isolation, the individual fixed effects were also non-significant (days since first dehorning: *P* = 0.256; number of dehorning procedures: *P* = 0.979; Table 3; first dehorning: mean 0.948 ± 0.280 μg/g; second dehorning: 1.359 ± 0.408 μg/g).

The number of times a male rhino was dehorned influenced the rate of change in FAM concentrations over time, as shown by the significant interaction term (*P* = 0.002; Table 3). When considering the terms of the individual fixed effects, FAM concentrations significantly increased in the days since first dehorning (*P* < 0.001; Table 3) but were not influenced by the number of dehorning procedures (*P* = 0.135; Table 3; first dehorning: mean 0.262 ± 0.086 μg/g; second dehorning: 0.262 ± 0.107 μg/g; third dehorning: 0.369 ± 0.147 μg/g).

## Discussion

### Short-term physiological response (up to 1 week after dehorning)

The lack of a short-term hormone response to immobilization and dehorning runs counter to the trend observed by Badenhorst *et al.* (2016) whereby rhinos underwent a spike in FCM concentrations immediately after their dehorning, experiencing an acute endocrine stress response. The differences between the two studies may be explained by variation in the timings of sample collection. Badenhorst *et al.* (2016) reported a peak in FCMs 24–48 hours after dehorning, which rose to a median 32% (range: −7.0 to 293%) above the baseline, whereas by 72–86 hours after the event FCMs had decreased by a median of 16% (range: −24 to 42%) above the pre-dehorning baseline. In comparison, the samples in this study were collected a mean 80 hours (range: 24–166 hours) after dehorning and had declined a median 17.2% (range: −60 to 43%) below the pre-dehorning concentration. Thus, the sampling times of the current study will have missed the peak period of FCM excretion if it occurred soon after the dehorning procedure.

Additionally, it remains possible that the rhinos in this study did not undergo an increase in FCMs following dehorning. The difference between our findings and those of Badenhorst *et al.* (2016) could relate to how one population was free-ranging and the other was intensively reared. The adrenocortical activity of animals kept under different management conditions can vary substantially, for example, captive white rhinos kept under differing housing conditions can show significantly different FCM concentrations, while the FCMs of captive born individuals can differ from those of wild caught individuals (Metrione and Harder, 2011). Whether a stress-induced FCM peak was truly absent or occurred prior to sample collection, the physiological effects of dehorning did not extend to the week after the procedure.

### Longer-term physiological response (up to 142 days after dehorning)

FCM profiles of the recently dehorned group were no different to those animals that did not undergo the procedure. Thus, there is no evidence that immobilization during the horn removal procedure or any resultant behavioural changes caused by a reduction in horn mass induced a chronic physiological stress response. However, detrimental changes in behaviour may not necessarily manifest themselves as a detectable physiological response (Meister, 1998; Metrione and Harder, 2011) or alternatively may only occur under certain circumstances. For example, white rhinos kept at higher population densities can show greater levels of aggressive social behaviours (Metrione *et al.*, 2007) and atypical social structures to those kept at lower densities (Cinková and Bičík, 2013). Thus, there is a need to conduct behavioural follow-up studies to see if the effects of dehorning extend beyond a negligible physiological impact and extend across other conditions.

### Response to multiple dehorning procedures

The number of dehorning procedures did not influence rhino FCM profiles. It appears that repeat immobilizations along with the punctuated reductions in horn mass (~1 kg/dehorning) do not act as a long-term stressor. This corroborates the results of the above FCM analyses and extends the findings to repeat dehorning procedures (with subjects dehorned between one and three times each during the study). However, as the experimental design is correlative, it remains possible that other physiological or psychosocial stressors experienced over the course of the study may have occluded the detection of a significant effect (Kretzschmar *et al.*, 2004; Palme, 2019). For example, differences in reproductive state (Edwards and Boonstra, 2018) and changes in social behaviours (Carlstead and Brown, 2005) are known to affect animal glucocorticoid responses. In comparison, environmental stressors such as reduced availability of foraging resources (Parry-Jones *et al.*, 2016) are likely to have had a limited impact on the relationship as calendar date is not coincident with ‘Days Since First Dehorning’ as the subjects were not all dehorned on the same day.

The non-significant relationship between the number of dehorning procedures and rhino FPM profiles provides evidence that the effects of the procedures were minimal and did not lead to chronic stress. Management strategies that mitigate against chronic stress responses in wild animals have the greatest chances of increasing future reproductive success (Dickens *et al.*, 2010). Thus, the absence of suppression in progesterone metabolites observed in this study supports that dehorning can act as a practical anti-poaching tool without compromising reproductive performance. This is further supported by research from Penny *et al.* (2020) that reported that the average inter-calf intervals of female rhinos at the same study site did not increase following dehorning. Additionally, a study on an intensively reared population of dehorned white rhinos by Ververs (2018) failed to detect a change in the birth sex ratio of calves. Such a change has been observed in individuals subject to translocation with the direction of bias depending on the stage of gestation that a rhino was at during this practise (Linklater, 2007).

The number of dehorning procedures influenced the rate of change in FAM concentrations over time. Interestingly, FAM concentrations increased after the third dehorning, rather than an expected suppression if rhinos were experiencing chronic physiological stress. This may indicate a release from chronic stress if rhinos acclimatized to repeat procedures. There is no consensus for what constitutes a stereotypical hormone change in chronically stressed animals (Dickens and Romero, 2013); however, an increase in FCMs is usually anticipated (Palme, 2019). As this was not detected, the observed trends in FAM profiles are unlikely representative of chronic stress, unless FCMs peaked prior to the monitored period and then declined to pre-stressed concentrations (Linklater *et al.*, 2010). Either way, the potential negative effects on rhino reproductive health appear to be short-term, with FAM concentrations rising towards the end of the monitored period.

The lack of suitable control conditions for the multiple procedure analyses means that the apparent link between FAM profiles and dehorning requires further confirmatory research. A major limitation with this study is that the timings of repeat dehorning procedures were inseparable to the increasing age of the study subjects. Throughout the study, several subadults began to exhibit sexual behaviours, such as urine spraying and foot scraping (Owen-Smith, 1975). Sexual behaviours are a predictor of FAM concentrations in subadult rhinos (Ververs, 2018). Thus, the observed relationship may reflect developmental changes brought on by the onset of puberty and sexual maturity rather than those caused by dehorning. While cause and effect remain unclear, it appears that repeat procedures do not suppress FAM concentrations over the long term and are not an ongoing cause of physiological stress.

### Limitations of the study and future work

The inclusion of a larger number of fixed effects within the GLMMs would have been desirable but the sample size and correlations between variables of interest (e.g. ‘Days Since First Dehorning’ and ‘rhino age-class’) limited the complexity of the models. The relationship between age and white rhino FCM profiles requires further study, with Badenhorst *et al.* (2016) reporting a lower baseline concentrations in juvenile white rhinos than in adults but Brown *et al.* (2001) finding no age-related differences. The limitations of pooling subjects across different age groups are further highlighted by a study of African buffalo (*Syncerus caffer*), where adults but not subadults experienced a spike in FCMs in response to chemical immobilization (Spaan *et al.*, 2017).

Intra- and inter-assay variations may have occluded the detection of minor changes in hormone concentration. However, as FCMs of white rhinos may increase by two to five times in response to a stressor (Linklater *et al.*, 2010) assay precision is unlikely to have affected the detection of a significant effect. The previous study on dehorning by Badenhorst *et al.* (2016) utilized a group specific (5α-pregnane-3β,11β,21-triol-20-one) assay that can detect a wider range of FCMs than a standard corticosterone (4-pregnene-11ß,21-diol-3,20-dione) assay (Palme, 2019). However, as past studies of white rhino have successfully detected FCM peaks via corticosterone assays, the approach used here remains valid (Brown *et al.*, 2001; Linklater *et al.*, 2010). Such methodological variations complicate the comparisons of concentrations of hormone metabolites between studies and highlight the need for the use of standardized techniques (Dickens and Romero, 2013; Palme, 2019).

It would be beneficial if future longitudinal studies on rhino stress physiology controlled for life histories, resource availability, habitat type and anthropogenic disturbance, as all of these can influence the endocrine profiles of wildlife species (Creel *et al.*, 2002; Brown and Fuller, 2006; Goymann, 2012; Pokharel *et al.*, 2017; Webster *et al.*, 2018). The inclusion of non-dehorned control animals that undergo chemical immobilization but not dehorning would also help elucidate whether physiological changes relate to a decrease in horn size or the dehorning procedure itself. The inclusion of populations across multiple reserves would help distinguish site-specific factors. This could best be achieved through studies of captive or range-limited populations for which the logistics of sample collection and the standardization of conditions are less challenging (Palme, 2019), as a previous short-term study of rhinos demonstrated (Badenhorst *et al.*, 2016).

The absence of a detectable chronic stress response following dehorning means the technique compares favourably with other forms of rhino management, including translocation, a commonly used conservation intervention that can elevate physiological stress for several weeks (Linklater *et al.*, 2010; Yang *et al.*, 2019). These findings should provide confidence to conservation practitioners that the procedure can be used safely, with the caveat that further research is required into the effectiveness of dehorning as a long-term method to reduce poaching (Lindsey and Taylor, 2011) and into whether it impacts the behaviour of white rhinos, both of which were outside the scope of this study.

## Funding

This work was supported by a PhD studentship (to S.G.P.) and a grant from the Earthwatch Institute (P30U R0913).
